# Epidemiology of injuries in young volleyball athletes: a systematic review

**DOI:** 10.1186/s13018-023-04224-3

**Published:** 2023-10-04

**Authors:** André de Azevedo Sodré Silva, Luana Beatriz Sassi, Tamiris Beppler Martins, Fábio Sprada de Menezes, Filippo Migliorini, Nicola Maffulli, Rodrigo Okubo

**Affiliations:** 1 Department of Physiotherapy, University of the State of Santa Catarina, Florianópolis, SC Brazil; 2 Department of Orthopaedic, Trauma, and Reconstructive Surgery, RWTH University Medical Centre, Pauwelsstraße 30, 52074 Aachen, Germany; 3 Department of Orthopedics and Trauma Surgery, Academic Hospital of Bolzano (SABES-ASDAA), Teaching Hospital of the Teaching Hospital of Paracelsus Medical University, 39100 Bolzano, Italy; 4 Department of Trauma and Orthopaedic Surgery, University of SalernoUniversity Hospital Sant’ Andrea, University La Sapienza, 00185 Rome, Italy; 5 School of Pharmacy and Bioengineering, Faculty of Medicine, Keele University, Stoke on Trent, ST4 7QB UK; 6 Centre for Sports and Exercise Medicine, Barts and the London School of Medicine and Dentistry, Mile End Hospital, Queen Mary University of London, London, E1 4DG UK; 7 Physical Therapy Graduate Program, University of the State of Santa Catarina, Florianópolis, SC Brazil

**Keywords:** Epidemiology, Athletes, Volleyball, Young, Juvenile

## Abstract

**Background:**

Volleyball is among the five most popular sports in the world. Regardless of level and age, volleyball athletes perform fast high-impact movements such as jumps, landings, and changes in direction, demanding motor and sensory skills to avoid injuries. The available scientific literature provides information regarding the incidence of injuries in volleyball, but the evidence of injuries in young volleyball athletes (12–18 years old) is not well defined. Therefore, a systematic review was conducted to investigate the incidence and prevalence of injuries in young volleyball players.

**Methods:**

This systematic review was conducted according to the PRISMA recommendations and prospectively registered in PROSPERO (ID: CRD42022344623). An electronic search was conducted in the following databases: Web of Science, PubMed, and SportDiscuss via EBSCO in August 2022 and March 2023. Inclusion criteria followed the PICOS acronym: (P) youth volleyball players; (I) volleyball; (C) none; (O) incidence and/or prevalence of injury; and (S) cohort studies. The risk of bias was analysed using the adapted STROBE instrument.

**Results:**

Five studies were included in the qualitative analysis. They had a mean methodological quality of 6 (range 4–8) on the modified STROBE scale. Injury incidence was presented in varying ways, ranging from 1.51 injuries/1000 player hours to 12.4 injuries/10,000 athlete exposures (AEs). The prevalence was 1.6 ± 1.7 per 100 AEs. A total sample of 3698 youth volleyball athletes predominantly females was found. The body sites with the highest rate of injuries were the ankle, the distal portion of the upper limbs (wrist/hand/fingers) and the knee, respectively.

**Conclusion:**

There was remarkable variability in the rate of injuries and the form of presentation between the studies. In addition, junior volleyball athletes had lower injury rates compared to other sports practised in high school, and older athletes had higher injury rates.

## Introduction

The sport of volleyball was formalised in 1895, showing exponential growth over the decades, with one of the highest participation rates of any sport in the world [1]. In addition, the sport has categories that vary in age and competition level. Regardless of the level and age, during sports practice, volleyball athletes perform fast movements such as jumps, landings, and changes of direction, with demanding motor and sensory skills necessary to avoid injuries [2].

Injuries occurring in sports can remove athletes from training and competitions, with undue consequences to individuals and the club [3], making it necessary to understand the frequency and location of injuries [4]. The prevalence of musculoskeletal injuries in volleyball reaches 10.7 injuries every 1000 h played [5]. Injuries occur in the ankle, knee and shoulder, with the highest incidence of acute injuries in the ankle, and overuse injuries more frequent in the shoulder and knee [6].

Many volleyball players start young [7]. In countries such as the USA, approximately 27 million young people aged 6–8 years participate in team sports [8]. Even with the positive effects that sport provides in adolescents [9], the risks of injuries should be acknowledged [10].

Comparing primary school and high school athletes, the difference in the general rates of injuries and environments, with a higher number of matches and training sessions in high school athletes, is evident [11]. Marked differences in injuries were reported in high school and university students, with variations both in the general rate and in the type of injury [10].

Although volleyball has a low number of injuries compared to other sports [12–14], tools are needed to quantify these injuries, thus providing a better approach to strategies to reduce injury rates [15]. These strategies include effective preventive measures, recognising the need to know the incidence and aetiology (risk factors and mechanisms) of lesions to promote appropriate preventive actions for a specific population [16].

Although the literature provides some information regarding the incidence of injuries in volleyball [11], the related evidence in young athletes (12–18 years old) is not well defined. This study analyses the incidence and prevalence of injuries, population profile and level of practice in young volleyball athletes.

## Material and methods

### Eligibility criteria

This review included studies that evaluated the incidence and prevalence of injuries among young volleyball athletes. Eligible studies had to describe injury evaluation and the mean age of the participants. All published cohort studies that investigated the incidence of injuries in youth volleyball athletes were included. Comments, reviews, case reports, editorials, letters to the editor, and technical notes were not eligible. Only articles in English were considered. Only studies that reported quantitative data regarding injury incidence were included.

### Search strategy

This systematic review was conducted according to the preferred reporting items for systematic reviews and meta-analyses (PRISMA) recommendations [17, 18] and prospectively registered in PROSPERO (ID: CRD42022344623). The electronic search was conducted in the following databases: Web of Science, PubMed, and SPORTDiscus via EBSCO, in August 2022 and March 2023.

The search keywords were determined through the acronym PICOS:

- P (population): volleyball players aged 12 and 18 years.
- I (intervention): volleyball.
- C (control): none.
- (outcome): incidence and/or prevalence of lesions.
- S (studies): cohort studies.

The Medical Subject Headings (MeSH) terms in English used were: ((volleyball) OR ("volleyball athletes") OR (volley)) AND ((Injury) OR ("common injuries") OR (injuries) OR (prevalence) OR (incidence)).

### Screening and data extraction

Study selection was conducted by two reviewers (**;**) independently. The eligibility criteria were first used to exclude titles, then abstracts, and finally full articles. The bibliography of the included studies was also screened by hand. The selection was performed using Microsoft Office Excel version 16.72 (Microsoft Corporation, Redmond, USA). If no consensus was reached between the two reviewers, a third reviewer (**) was consulted.

Data extraction also was conducted by two reviewers (**;**) independently. Injury incidence rates of hours of player exposure were extracted from the included studies. Data were extracted using Microsoft Office Excel version 365 (Microsoft Corporation, Redmond, USA). If no consensus was reached, a third reviewer (**) was consulted.

### Methodological quality and data synthesis

The methodological quality assessment was carried out by the two reviewers (AS and LS), using the modified STROBE classification, which consists of a checklist consisting of 11 items, which was used in a previous study by López-Valenciano [19]. In the scale, are evaluated aspects regarding the methods and results of each included study.

Additionally, to assess the risk of bias of external validity quality, an adapted version of the Newcastle–Ottawa Scale (NOS) for cohort studies was used [19]. The adapted criteria to assess the risk of bias were: (1) Description or type of volleyball players. (2) Definition of injury. (3) Representativeness of the exposed cohort. (4) Ascertainment of exposure. (5) Demonstration that the outcome of interest was not present at the start of the study. (6) Assessment of outcome. (7) Whether follow-up was long enough for outcomes to occur. (8) Adequacy of follow-up of cohorts. Each study could be awarded a maximum of one star for each item if appropriate methods had been reported.

## Results

### Study selection

**Fig. 1:**
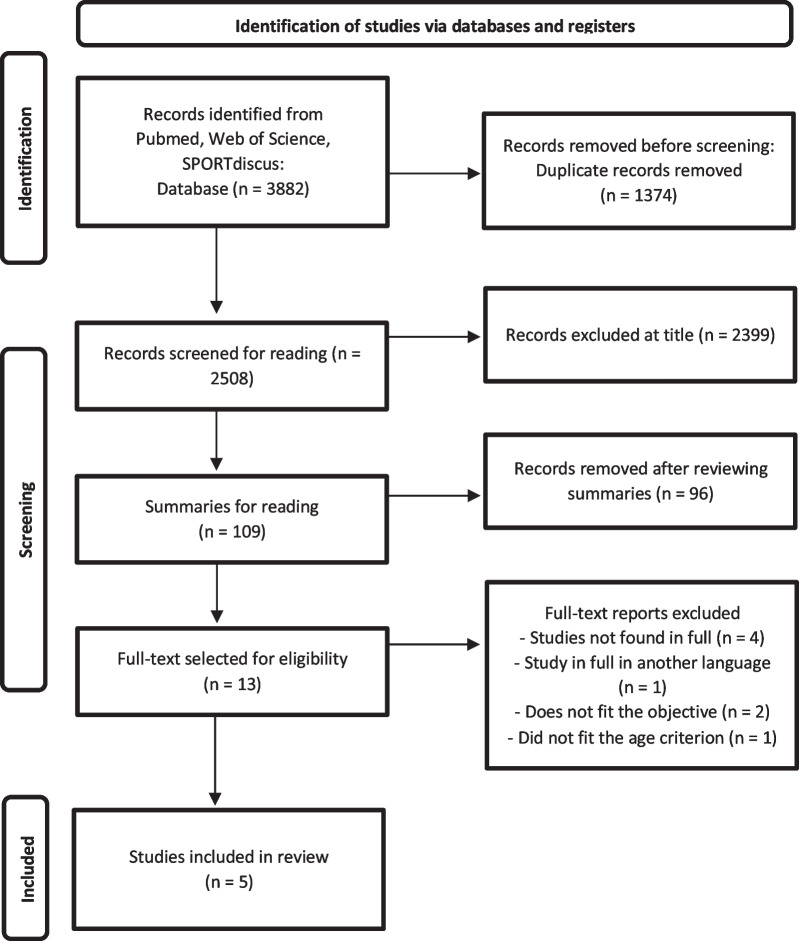
Flowchart of the search process

The electronic search in the databases resulted in 3882 results: 1410 in Web of Science, 1291 in SPORTDiscus and 1181 in PubMed (Fig. 1). After removing duplicates, 2508 titles remained. Then, after reading the titles, 109 abstracts remained. After reading the abstracts, 13 complete articles were selected for eligibility. Finally, 5 (five) articles were included in the methodological quality assessment and qualitative data analysis [10, 20–23].

### Characteristics of included studies

The selected studies included a total of 3698 athletes. The age of the participants ranged from 12 to 18 years, with a mean age of 15.4 years, with the vast majority being female (91.4%). Participants per study ranged from 36 to 2072. Four studies were retrospective, and one was conducted prospectively (Table 1).

**Table 1:** Methodological quality analysis of selected studies—STROBE (*n* = 5)

Study	1	2	3	4	5	6	7	8	9	10	11	Score
Azuma et al. [20]	+	+	+	−	−	−	+	+	+	−	+	7
McGuine et al. [21]	+	+	+	+	+	−	+	−	+	−	+	8
Reeser et al. [10]	+	+	+	−	−	−	+	−	−	−	+	5
Vanderlei et al. [22]	+	−	+	−	−	−	+	−	−	−	+	4
Wasser et al. [23]	−	−	+	−	−	−	+	−	+	+	+	5

(1) Describes the setting or participating locations

(2) Describes relevant dates (period of recruitment, exposure, follow-up, data collection)

(3) Provides statement concerning institutional review board approval and consent

(4) Gives the inclusion and exclusion criteria

(5) Describes injury history

(6) Describes methods of follow-up

*Data sources/measurement*

(7) Provides a definition of injury

(8) Verifies injury by an independent medical professional

(9) Classifies injury (severity, location and type of injury)

(10) Indicates the number of participants with missing data and explain how this was addressed

(11) Measures and presents exposure data

### Quality of included studies

Tables 2 and 3 highlight the variability among the selected articles when evaluated by the modified STROBE and adapted NOS instruments. For the STROBE, on a numeric scale from 0 to 11, the article that had the highest score reached 8 (eight) points [21] and the lowest 4 (four) points [22]. The average score, considering all included studies, was 6 (ranging from 4 to 8 points). For the adapted NOS, three articles reached the highest score of eight points [6, 17, 19] and two articles scored five [16, 20]. The average score was 6.8 points.

**Table 2:** Methodological quality analysis of selected studies—STROBE (*n* = 5)

Study	1	2	3	4	5	6	7	8	9	10	11	Score
Azuma et al. [17]	+	+	+	−	−	−	+	+	+	−	+	7
McGuine et al. [19]	+	+	+	+	+	−	+	−	+	−	+	8
Reeser et al. [6]	+	+	+	−	−	−	+	−	−	−	+	5
Vanderlei et al. [16]	+	−	+	−	−	−	+	−	−	−	+	4
Wasser et al. [20]	−	−	+	−	−	−	+	−	+	+	+	5

(1) Describes the setting or participating locations

(2) Describes relevant dates (period of recruitment, exposure, follow-up, data collection)

(3) Provides statement concerning institutional review board approval and consent

(4) Gives the inclusion and exclusion criteria

(5) Describes injury history

(6) Describes methods of follow-up

*Data sources/measurement*

(7) Provides a definition of injury

(8) Verifies injury by an independent medical professional

(9) Classifies injury (severity, location and type of injury)

(10) Indicates the number of participants with missing data and explain how this was addressed

(11) Measures and presents exposure data

**Table 3:** Risk of bias of external validity quality of selected studies—NOS (Newcastle–Ottawa scale) adapted (*n* = 5)

Study	1	2	3	4	5	6	7	8	Score
Azuma et al. [17]	+	+	+	+	+	+	+	+	8
McGuine et al. [19]	+	+	+	+	+	+	+	+	8
Reeser et al. [6]	+	+	+	+	+	+	+	+	8
Vanderlei et al. [16]	+	+	+	+	−	+	−	−	5
Wasser et al. [20]	+	+	+	+	−	+	−	−	5

Criteria for assessing risk of bias

(1) Description or type of volleyball players

(2) Definition of injury

(3) Representativeness of the exposed cohort

(4) Ascertainment of exposure

(5) Demonstration that outcome of interest was not present at start of study

(6) Assessment of outcome

(7) Was follow-up long enough for outcomes to occur

(8) Adequacy of follow-up of cohorts

+Plus awarded for each criterion

### Incidence/prevalence of injuries

The distribution of injury incidences was 1.51 injuries per 1000 h per player [17], 5.3 injuries per 1000 AEs [19], 12.4 injuries per 10,000 AEs (1.24/1000 AEs) [6], 0.237 injuries/participant [16] and 1.19 injuries/injured participant [20]. Only one study described the prevalence of 1.6 ± 1.7 per 100 AEs (16.0/1000 AEs) (Table 1). An explanation of what would be considered as incidence and prevalence of injuries is reported in Table 4.

**Table 4:** Form of presentation of incidence and prevalence

Study	Form of presentation	Definition
Azuma et al. [17]	1.000 h per player	Equivalent to injuries incurred by each player every 1000 h played by them
McGuine et al. [19]	1.000 AEs	AEs: Any event, whether training or championship directed by the coach
Reeser et al. [6]	10.000 AEs	AEs: participation of 1 athlete in training or competition regardless of duration
Vanderlei et al. [16]	Injury/player	Calculated an absolute number of injuries per participant, regardless of the number of exposures/hours played
Wasser et al. [20]	100 AEs	AEs: game or practice 2 h long

*AEs athletic exposures

### Characteristics of injuries and associated factors

The definition, characteristics and associated factors of the injuries, per each study, are detailed in Table 5. The associated factors associated with a higher rate of injuries were: older age; previous history of musculoskeletal injury; higher body mass index (BMI); greater sports participation during the year; and play in the libero position.

**Table 5:** Characteristics of injuries

References	Injury site (%)	Associated factors and time of injury	Definition of injury
Azuma et al. [17]	Ankle (20.6)	Trauma during competition (+)*	Condition acquired from participation in training or competition that results in removal from the same activities for at least 1 day
	Hand/wrist/fingers (17.6)		
	Knee (14.7)		
McGuine et al. [19]	Ankle (23.5)	Older players	Acute or gradual injury resulting from participation in volleyball training or competition and requiring attention from a health professional
	Hand/wrist/fingers (16.2)	Higher body mass index (BMI) (+)	
	Knee (14.4)	Time loss 2× > Non time loss injury	
Reeser et al. [6]	Ankle (36.6)	History of musculoskeletal injury sports ** (+)	Condition acquired from participation in training or competition that results in removal from the same activities for at least 1 day
	Hand/wrist (12.2)		
	Knee (10.9)		
Vanderlei et al. [16]	Ankle (36.3)	Older age > 15.5 years (+)	Musculoskeletal condition with signs and symptoms arising from sports practice, which compromise training/championship in terms of quality, duration, intensity or frequency
	Hand/wrist (20.1)	Higher BMI > 66 kg (+)	
	Knee (15.3)	Higher height > 1.74 cm (+)	
Wasser et al. [20]	Ankle (40.6)	Greater sports participation during the year, 9.4 × 8.4 months (+)	Condition acquired from participation in training or competition that results in removal from the same activities for at least 1 day
	Fingers (36.6)		
	Knee (21.2)	Libero position (+)	

*(+) higher injury rate

**For injuries with + 1 day loss of participation

***Age in years

Three studies used the same definition of injury, namely a condition acquired from participation in training or competition that resulted in absence from these activities for at least 1 day [10, 20, 23]. The form of presentation of incidence and prevalence of injuries was presented differently in each study. Moreover, prevalence was addressed in only one article [23].

## Discussion

This systematic review aimed to verify the epidemiology of injuries, the population and injury profile, and the level of practice of young volleyball athletes. Injury incidences ranged from 1.24 injuries/1000 h per player [20] to 5.3 injuries/1000 athlete exposures (AEs) [10]. Consistent with the findings of this systematic review, compared to eight other sports practised by junior high school athletes in the USA, women's volleyball is one of the sports with the lowest injury rates, both acute 9.9/10,000 AEs and overuse 1.5/10,000 AEs [24]. Agel et al. reported an overall injury rate of 4.58 per 1000 athlete exposures (AEs) during games and 4.1 injuries per 1000 AEs during practice in the period from 1988 to 2004 [25]. We remark on the high prevalence rate recorded by the only study that considers such data: the values were three times higher (16.0/1000 AEs) than the highest incidence of the analysed studies.

Reeser et al. [10] compared junior high school volleyball athletes with university volleyball athletes, showing that university athletes had an injury rate three times higher. When making a comparison with the adult population (total injury incidence 1.7 ± 0.2 per 1000 h of play, 1.5 ± 0.2 during training and 3.5 ± 0.8 during match play) [8, 26], youth athletes experience lower rates of injuries. The factors associated with higher injury rates in advancing age were the high level of competition, and increasing susceptibility to injuries [27]. Also, previous injuries increase the risk of further injuries [28].

Regarding the anatomical location of the injuries, in accordance with other studies, all articles included in the present investigation reported the ankle as the most common body part, followed by the wrist, hand, fingers, and knee [8, 29]. Volleyball is not a contact sport, but most ankle sprains occur from landing from a jump on the foot of another athlete [11].

Some of the associated factors which lead to a higher injury rate were older age; previous history of musculoskeletal injury; taller stature; greater body mass; higher BMI; greater sports participation during the year; and play in the ‘libero’ position. The age factor [30, 31] and higher BMI [32] are in line with previous studies, given the increased competition level and overload with age and greater overload on joints with higher BMI. In addition, regarding the previous musculoskeletal injury factor, it is in line with the main injury, since previous ankle sprains are a risk factor for further sprains [33].

Haupental et al. reported an incidence of seven injuries/1000 h of competition and two injuries/1000 h of training. The regions with the highest prevalence of injuries were the knee (111/1000 athletes) and ankle (69/1000 athletes). Athletes aged above 23 years and those playing as middle blockers and outside hitters presented a higher prevalence of injuries [34].

Female athletes (91.6%) greatly predominate over male athletes (8.4%). This is possible because most of the articles included in the analysis originated from the USA, where volleyball is very popular among females, both at school and university [35].

The average STROBE score was 6 (ranging from 4 to 8 points). The difficulties found in the studies were in items 4, 5, 6, 8 and 10 regarding the inclusion and exclusion criteria, describing injury history, to describe methods of follow-up, to verify injury by an independent medical professional to indicate the number of participants with missing data. However, as the analysis was performed using an observational tool to analyse bias, the quality of the evidence has a greater tendency to be high. As for NOS, the mean value was 6.8 points. The most important limitations highlighted by the NOS were at points 5, 7 and 8, indicating flaws in the definition of the results of interest and the inadequacies of the follow-up. However, these were typical of retrospective studies which asked athletes about their injuries. Additional limitations were evident, including the heterogeneity of the included studies, mainly regarding the presentation of the incidence and prevalence indices (number of AEs, hours played by each player, etc.), and the variability in data collection modalities (for coaches, athletes, software, and authors themselves). The follow-up periods varied considerably among the studies (ranging from 1 month to 4 years). Studies with shorter follow-up periods may have more significant limitations, which should be considered when interpreting and extrapolating the results. Moreover, the reported injuries in the studies varied between hours per player, athletic exposures and injuries per participant: these different methods of reporting injuries may have influenced the observed injury rates. It is also important to point out that only one study reported information regarding the prevalence of injuries, and therefore, this value has to be interpreted with caution. In addition, we caution that a total of five studies were included in the analysis, as volleyball in younger has been seldom studied.

## Conclusion

The prevalence and incidence of injuries in young volleyball athletes were low. The injury rate is relatively low when compared to other commonly practised sports in high school and lower than in older volleyball players. In addition, the most commonly affected regions were the ankle, upper limb (wrist, hand and fingers) and knee. Finally, the factors associated with the occurrence of injury include older age, previous history of musculoskeletal injury, higher BMI, and higher frequency of sports participation during the sporting year.

## Data Availability

The datasets generated during and/or analysed during the current study are available throughout the manuscript.

## Declarations

### Registration and protocol

PROSPERO registration ID: CRD42022344623.
